# Prognostic factors associated with the transition in treatment methods for brain metastases from colorectal cancer

**DOI:** 10.1007/s10147-023-02352-8

**Published:** 2023-05-19

**Authors:** Jun Imaizumi, Dai Shida, Narikazu Boku, Hiroshi Igaki, Jun Itami, Yasuji Miyakita, Yoshitaka Narita, Atsuo Takashima, Yukihide Kanemitsu

**Affiliations:** 1 Department of Colorectal Surgery, National Cancer Center Hospital, 5-1-1 Tsukiji, Chuo-ku, Tokyo, 1040045 Japan; 2 Division of Frontier Surgery, The Institute of Medical Science, The University of Tokyo, 4-6-1 Shirokanedai, Minato-ku, Tokyo, 1088639 Japan; 3 Gastrointestinal Medical Oncology Division, National Cancer Center Hospital, 5-1-1 Tsukiji, Chuo-ku, Tokyo, 1040045 Japan; 4 Department of Oncology and General Medicine, IMSUT Hospital, The Institute of Medical Science, The University of Tokyo, Tokyo, Japan; 5 Department of Radiation Oncology, National Cancer Center Hospital, 5-1-1 Tsukiji, Chuo-ku, Tokyo, 1040045 Japan; 6 Department of Radiology, Shin Matsudo Central General Hospital, 1-380 Shinmatsudo, Matsudo-shi, Chiba 2700034 Japan; 7 Department of Neurosurgery and Neuro-Oncology, National Cancer Center Hospital, 5-1-1 Tsukiji, Chuo-ku, Tokyo, 1040045 Japan

**Keywords:** Brain metastases, Colorectal cancer, Karnofsky performance status, Stereotactic radiotherapy, Whole-brain radiotherapy

## Abstract

**Background:**

Treatment of brain metastases (BMs) from colorectal cancer (CRC) has transitioned with the expansion of indications for stereotactic radiotherapy. Our study aimed to assess changes in prognosis and prognostic factors associated with changes in treatment for BMs from CRC.

**Methods:**

We retrospectively surveyed treatments for and outcomes of BMs from CRC in 208 patients treated during 1997–2018. Patients were divided into two groups according to time of BM diagnosis, i.e., 1997–2013 (“first period”) and 2014–2018 (“second period”). We compared overall survival between the periods and assessed how the transition impacted prognostic factors affecting overall survival, including the following prognostic factors such as Karnofsky performance status (KPS), volume-related factors (BM number and diameter), and BM treatment modalities as covariates.

**Results:**

Of the 208 patients, 147 were treated in the first period and 61 in the second period. Whole-brain radiotherapy use decreased from 67 to 39% in the second period, and stereotactic radiotherapy use increased from 30 to 62%. Median survival after BM diagnosis improved from 6.1 to 8.5 months (*p* = 0.0272). Multivariate analysis revealed KPS, control of primary tumor, stereotactic radiotherapy use, and chemotherapy history as independent prognostic factors during the entire observation period. Hazard ratios of KPS, primary tumor control, and stereotactic radiotherapy were higher in the second period, whereas prognostic impact of chemotherapy history before BM diagnosis was similar in both periods.

**Conclusion:**

Overall survival of patients with BMs from CRC improved since 2014, which can be attributed to advances in chemotherapy and the more widespread use of stereotactic radiotherapy.

## Introduction

The most common intracranial tumors are metastases to the brain from lung cancer, breast cancer, renal cancer, and melanoma [1, 2]. The incidence of brain metastases (BMs) from colorectal cancer (CRC) is reported to be low, ranging from 1 to 4% [2–4], but may increase owing to improved diagnostic modalities and prolonged prognosis of metastatic CRC due to advances in systemic chemotherapy.

Using validated recursive partitioning analysis (RPA), the Radiation Therapy Oncology Group (RTOG) identified Karnofsky performance status (KPS) score, age, control status of the primary tumor, and extracranial metastases as prognostic factors for BMs from various types of cancers [5, 6]. In a diagnosis-specific graded prognostic assessment (DS-GPA), the number of BMs was added to the four prognostic factors used in the RPA model and validated in non-small cell lung cancer, breast cancer, renal cell carcinoma, melanoma, and gastrointestinal cancer cases [7, 8]. However, in the DS-GPA model, only KPS was considered a prognostic factor specific to primary gastrointestinal cancers, including CRC, gastric cancer, and esophageal cancer [7, 8].

Standard treatments for BMs are surgery and radiation therapy. The section on BMs in the 2019 Japanese Society for Cancer of the Colon and Rectum guidelines for treating CRC [9] states, ‘At present, whole-brain radiation therapy (WBRT) is performed irrespective of the number of metastases.’ However, WBRT is associated with late adverse events, including leukoencephalopathy, brain atrophy, and cognitive impairment [10, 11]. Stereotactic radiotherapy (SRT), which is usually indicated for tumors < 3 cm in diameter, offers clinical benefits over WBRT, including less toxicity and a shorter treatment time. In the JLGK0901 study conducted in 2014 [12], SRT without WBRT in patients with 5–10 BMs was non-inferior to that in patients with 2–4 BMs. This expanded the indication of SRT for up to 10 BMs. The NCCTG N0574 trial compared SRT and SRT + WBRT in patients with 1–3 BMs < 3 cm and found that cognitive decline was more frequent in the SRT + WBRT group than in the SRT group, although overall survival did not differ between groups [13]. Furthermore, in the JCOG0504 study, salvage SRT was non-inferior to WBRT and is now established as the standard treatment for patients with ≤ 4 BMs [14]. These clinical trials from around 2014 provide therapeutic evidence supporting the use of SRT to treat BMs [15]. In non-surgical cases, SRT has been the standard of care for up to 10 lesions since around 2014, and in surgical patients, salvage SRT has been the standard of care for up to 4 lesions since 2016.

Given the expanded indications for SRT, the National Comprehensive Cancer Network Clinical Practice Guidelines in Oncology of Central Nervous System Cancers, 2021 edition, [16] updated the definition of extensive or multiple BMs to reflect the concept of total intracranial tumor volume, while the number of intracranial lesions was added as an essential prognostic factor in the selection of treatment for BMs in the 2017 edition. However, the 2019 Japanese Society for Cancer of the Colon and Rectum guidelines [9] do not mention any changes to treatment options despite the expansion of indications for SRT. The present study aimed to assess changes in prognosis and prognostic factors associated with the transition in treatment methods for BMs from CRC.

## Patients and methods

### Subjects

Subjects of this retrospective study were patients with synchronous or metachronous BMs from CRC who were treated at the National Cancer Center Hospital (NCCH) between January 1997 and December 2018 and patients with BMs from CRC who underwent initial treatment for primary CRC at other institutions during the same period.

### Methods

Information on patients with BMs regarding sex, age, KPS score, number of BMs, maximum diameter of BMs, treatment for BMs, interval between diagnosis of primary CRC and BMs, location of primary tumor, presence of extracranial metastases at diagnosis of BMs, control status of the primary tumor, and history of systemic chemotherapy before the diagnosis of BMs was collected. The study period was divided into 1997–2013 and 2014–2018 based on when the results of the JLGK trial were published and led to the expansion of indications for SRT to up to 10 BMs [13].

### Ethical considerations

This study was approved by the Institutional Review Board (IRB) of the National Cancer Center Hospital (IRB code: 2017–437). The requirement for informed consent from each patient was waived owing to the study’s retrospective nature.

### Statistical analysis

OS was defined as the interval between the date of diagnosis of BMs and death from any cause or the date of last follow-up for patients who were censored. OS was estimated using the Kaplan–Meier method and compared using the log-rank test. Prognostic factors of OS were explored using multivariable Cox proportional hazards regression models, with the following factors at diagnosis of BMs included as covariates: age, KPS score, presence of extracranial metastases, and resection of primary tumor (all of which were previously reported prognostic factors for BMs), eligibility for SRT by size and number of BMs, history of systemic chemotherapy before diagnosis of BMs, use of targeted molecular therapy during the entire course of the disease, and modality of radiation for BMs with or without SRT and WBRT. For volume-related factors, 1–3 lesions with a maximum diameter of < 4 cm were defined as "limited,” and all other lesions were defined as "extensive.” Data are presented as the number of patients, ratio (%), hazard ratio (HR), and 95% confidence intervals (CIs). All statistical analyses were performed using JMP14.0.0 software (SAS Institute Japan Ltd., Tokyo, Japan). *p* < 0.05 was considered statistically significant.

## Results

### Patient demographics and clinical characteristics

**Fig. 1:**
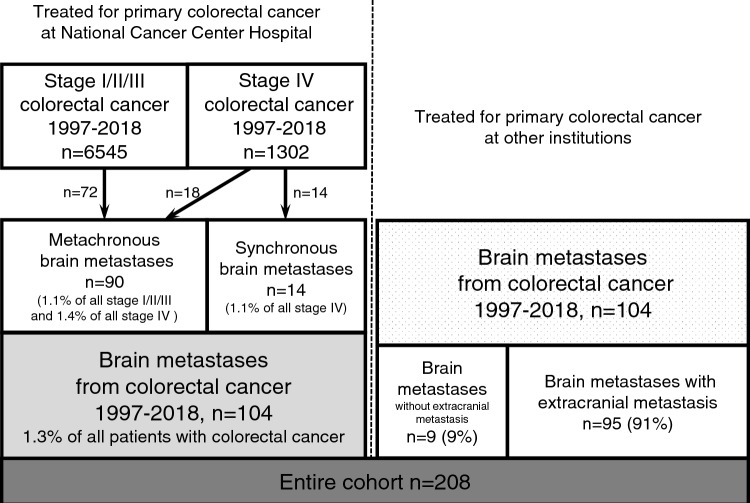
Flow chart for patient selection. The final study population consisted of 208 patients with brain metastases (BMs) from colorectal cancer (CRC) treated at the National Cancer Center Hospital (NCCH) from 1997 to 2017. Of these, 103 who underwent initial treatment for CRC at the NCCH accounted for 1.3% of all patients with CRC during the study period. The remaining 105 patients were referred to the NCCH for BMs after treatment of CRC at another hospital

During the study period, 6545 patients had stage I/II/III CRC and underwent primary tumor resection, and 1302 received treatment for stage IV CRC at NCCH. Fourteen (1.1%) patients with stage IV CRC developed synchronous BMs, and 90 developed metachronous BMs. In addition, 104 patients were referred to NCCH for the treatment of BMs after treatment for CRC at other institutions between 1997 and 2017. The final study cohort consisted of 208 consecutive patients (124 male, 84 female) with BMs. The CONSORT diagram is provided in Fig. 1.

Patient characteristics are shown in Table 1. The median interval between diagnosis of primary CRC and BMs was 2.7 years (interquartile range, 1.5–4.5); the interval was less than 3 years in 112 patients (53.8%). The median age at diagnosis of BMs was 63 years (interquartile range, 54–70). The KPS score was ≥ 70 in 118 patients (56.7%) and < 70 in 90 (43.3%). Extracranial disease was present in 185 patients (88.9%). Twenty-five patients (12%) had unresected primary tumors. Before the diagnosis of BMs, 167 patients (80.3%) had received systemic chemotherapy. There were 150 patients (72.1%) with 1–3 BMs and 58 (27.9%) with ≥ 4 BMs. Eighty-one patients (39%) had BMs > 3 cm in maximum diameter.

**Table 1:** Patient demographics and tumor characteristics

Variable						
Category	Entire cohort		1997–2013		2013–2018	
	*n* = 208		*n* = 147		*n* = 61	
	*n*	%	*n*	%	*n*	%
Gender						
Male	124	(59.6)	89	(60.5%)	35	(57.4%)
Female	84	(40.4)	58	(39.5%)	26	(42.6%)
Age at diagnosis of BMs, years	63	(54–69)	63	(54–70)	61	(52–68)
KPS score						
≥ 70	118	(56.7)	86	(58.5)	32	(52.5)
< 70	90	(43.3)	61	(41.5)	29	(47.5)
Presence of extracranial metastases						
Absent (brain only)	23	(11.1)	20	(13.6)	3	(4.9)
Present (brain and other sites)	185	(88.9)	127	(86.4)	58	(95.1)
Control of primary tumor						
Yes	183	(88.0)	132	(89.8)	51	(83.6)
No	25	(12.0)	15	(10.2)	10	(16.4)
BMs, *n*						
1	88	(42.3)	65	(44.2)	23	(37.7)
2	41	(19.7)	23	(15.6)	18	(29.5)
3	21	(10.1)	14	(9.5)	7	(11.5)
Extensive	58	(27.9)	45	(30.6)	13	(21.3)
Maximum diameter of BMs, mm	25	(16–33)	25	(16–32)	23	(14.5–35)
Volume-related factor (number and diameter of BMs)						
Limited (1–3 and < 4 cm)	127	(61.1)	87	(59.2)	40	(65.6)
Extensive (other than limited BMs)	81	(38.9)	60	(40.8)	21	(34.4)
History of systemic chemotherapy before BMs						
0	41	(19.7)	28	(19.0)	13	(21.3)
1	57	(27.4)	43	(29.3)	14	(23.0)
2	45	(21.6)	35	(23.8)	10	(16.4)
3	36	(17.3)	29	(19.7)	7	(11.5)
4	16	(7.7)	10	(6.8)	6	(9.8)
≥ 5	14	(6.7)	2	(1.4)	11	(18.0)
Use of target agent						
Not used	127	(61.1)	108	(73.5)	19	(31.1)
Used	79	(38.0)	39	(26.5)	40	(65.6)
Unknown	2	(1.0)	0	(0.0)	2	(3.3)
Location of primary tumor						
Colon	127	(61.1)	62	(59.6)	65	(62.5)
Rectum	81	(39.0)	42	(40.4)	39	(37.5)
Time interval from diagnosis of primary tumor to BMs, years						
< 3	112	(53.8)	87	(59.2)	25	(41.0)
≥ 3	95	(45.7)	59	(40.1)	36	(59.0)

*BMs* brain metastases, *KPS* Karnofsky performance status, *SRT* stereotactic radiotherapy, *WBRT* whole-brain radiotherapy

^a^Data presented as median (interquartile range)

### Transition in treatment methods

**Fig. 2:**
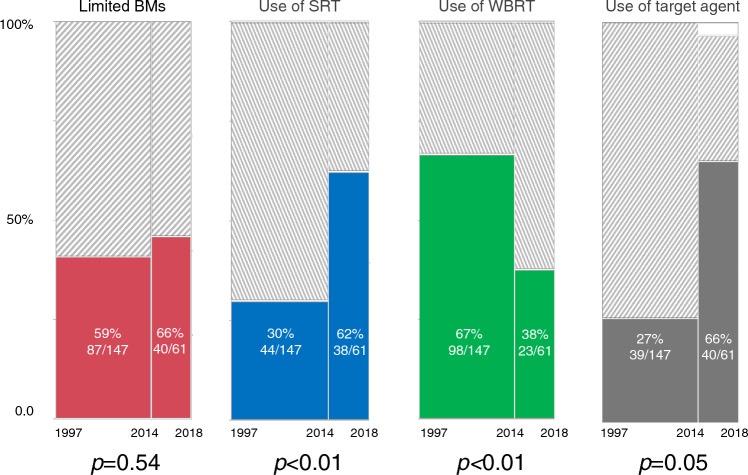
Transition in proportion of patients with limited brain metastases and treatment methods. The study population was divided into two study periods according to the time of diagnosis of brain metastases. In the first period, more than half of the patients received whole-brain radiotherapy (WBRT), and less than half received stereotactic radiotherapy (SRT). By the final study period, the proportion of patients who received WBRT decreased to 38% and the proportion receiving SRT increased to 62%, with a noticeable change in the proportion of patients with limited brain metastases

As mentioned above, the time of diagnosis of BMs was divided into two time periods: 1997–2013 (“first period”; *n* = 127) and 2014–2018 (“second period”; *n* = 61). Table 2 shows the treatment methods for BMs during each period, and Fig. 2 shows the transition in these methods. In the first period, more than half of the patients received WBRT (66.7%), but this significantly decreased to 39.3% (*p* = 0.0001) in the second period. The number of patients who received SRT significantly increased from 29.9% in the first period to 62.3% in the second period (*p* < 0.0001). For limited BMs (1–3 lesions < 4 cm in maximum diameter), the number of patients who received SRT increased significantly from 43% in the first period to 80% in the second period. Overall, there was a significant increase in the use of SRT and a decrease in the use of WBRT since 2014.

**Table 2:** Number of cases by treatment method

Treatment method	Entire cohort				Limited^1^ BMs				Extensive^2^ for SRT			
	1997–2013		2014–2018		1997–2013		2014–2018		1997–2013		2014–2018	
	*n* = 147		*n* = 61		*n* = 87		*n* = 40		*n* = 60		*n* = 21	
	*n*	%	*n*	%	*n*	%	*n*	%	*n*	%	*n*	%
SRT performed	44	(29.9)	38	(62.3)	38	(43.7)	32	(80.0)	6	(10.0)	6	(28.6)
SRT alone	30	(20.4)	23	(37.7)	28	(32.2)	23	(57.5)	2	(3.3)	0	(0.0)
SRT + WBRT	5	(3.4)	3	(4.9)	4	(4.6)	1	(2.5)	1	(1.7)	2	(9.5)
SRT + surgery	8	(5.4)	11	(18.0)	6	(6.9)	8	(20.0)	2	(3.3)	3	(14.3)
SRT + surgery + WBRT	1	(0.7)	1	(1.6)	0	(0.0)	0	(0.0)	1	(1.7)	1	(4.8)
SRT not performed	103	(70.1)	23	(37.7)	49	(56.3)	8	(20.0)	54	(90.0)	15	(71.4)
WBRT alone	71	(48.3)	10	(16.4)	34	(39.1)	2	(5.0)	37	(61.7)	8	(38.1)
WBRT + surgery	21	(14.3)	9	(14.8)	11	(12.6)	4	(10.0)	10	(16.7)	5	(23.8)
Surgery alone	4	(2.7)	3	(4.9)	2	(2.3)	2	(5.0)	2	(3.3)	1	(4.8)
Supportive care	7	(4.8)	1	(1.6)	2	(2.3)	0	(0.0)	5	(8.3)	1	(4.8)

^1^Limited: 1–3 lesions and < 4 cm in max diameter

^2^Extensive: other than the limited cases

### Overall survival after diagnosis of BMs

**Fig. 3:**
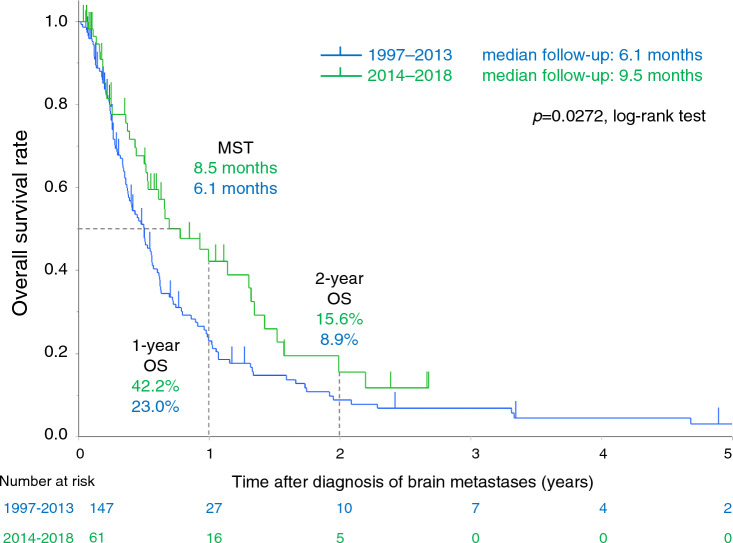
Overall survival curve for patients with brain metastases from colorectal cancer (*n* = 208). Median survival from the time of diagnosis of brain metastases was 6.2 months in the first period (1997–2013) and 8.5 months in the second period (2014–2018). The 1-year and 2-year survival rates were 23.0% and 8.9%, respectively, in the first period, and 42.2% and 15.6%, respectively, in the second period. *MST* median survival time, *OS* overall survival

OS and prognostic factors were compared between patients treated in the two periods. A survival curve for both periods is shown in Fig. 3. Median survival from the time of diagnosis of BMs was 6.2 months in the first period and 8.5 months in the second period (*p* = 0.0272). The 1-year and 2-year survival rates were 23.0% and 8.9%, respectively, in the first period, and 42.2% and 15.6%, respectively, in the second period.

### Prognostic factors after diagnosis of BMs

The overall Cox proportional hazards regression model results are shown in Table 3. Among all patients, independent prognostic factors were KPS score ≥ 70 [HR of KPS score < 70, 1.51 (95% CI 1.08–2.09); *p* = 0.0157], control of primary tumor [HR of uncontrolled, 1.79 (95% CI 1.05–2.92); *p* = 0.0331], SRT performed [HR of SRT not performed, 1.71 (95% CI 1.01–2.93); *p* = 0.0446], and no history of chemotherapy [HR of past history of systemic chemotherapy, 2.6, (95% CI 1.66–4.22); *p* < 0.0001].

**Table 3:** Multivariable analyses of factors affecting survival in patients with brain metastases from colorectal cancer

Variable	Category	Multivariable analysis								
		Entire cohort			1997–2013			2014–2018		
		*n* = 208			*n* = 147			*n* = 61		
		HR	95% CI	*p*-value	HR	95% CI	*p*-value	HR	95% CI	*p*-value
Age at time of BMs (years)	< 65	ref		0.9721	ref		0.689	ref		0.987
	≥ 65	1.01	0.69–1.45		0.92	0.59–1.40		1.01	0.41–2.37	
KPS score	≥ 70	ref		0.0157*	ref		0.143	ref		0.027*
	< 70	1.51	1.08–2.09		1.33	0.91–1.94		2.37	1.10–5.18	
Presence of extracranial metastases	Brain only	ref		0.1209	ref		0.057	ref		0.591
	Brain and other sites	1.55	0.90–2.81		1.85	1.01–3.57		0.62	0.12–4.53	
Control of primary tumor	Controlled	ref		0.0331*	ref		0.394	ref		0.011*
	Uncontrolled	1.79	1.05–2.92		1.32	0.67–2.45		3.99	1.40–10.7	
Volume-related factors	Limited (1–3 and < 4 cm)	ref		0.4068	ref		0.183	ref		0.898
	Extensive	1.16	0.81–1.66		1.32	0.88–1.99		0.93	0.32–2.69	
WBRT	Performed	ref		0.9667	ref		0.688	ref		0.801
	Not performed	0.99	0.59–1.64		0.88	0.47–1.61		1.16	0.36–3.63	
SRT	Performed	ref		0.0446*	ref		0.222	ref		0.208
	Not performed	1.71	1.01–2.93		1.51	0.79–2.95		2.16	0.66–7.61	
Targeted therapy	Performed	ref		0.06	ref		0.168	ref		0.44
	Not performed	1.4	0.50–1.00		1.35	0.89–2.10		1.39	0.54–3.47	
History of systemic chemotherapy before BMs	None	ref		< 0.0001*	ref		0.002*	ref		0.079
	Performed	2.6	1.66–4.22		2.4	1.38–4.31		2.34	0.91–6.86	

The significant *p*-values (*p* ≤ 0.05) are indicated with an asterisk (*)

*BMs* Brain metastases, *CI* confidence interval, *HR* hazard ratio, *KPS* Karnofsky performance status, *SRT* stereotactic radiotherapy, *WBRT* whole-brain radiotherapy

Comparing the two periods, HRs of the following factors were increased in the second period relative to the first period: KPS (HR from 1.33 to 2.37), controlled primary tumor (HR from 1.32 to 3.99), and SRT performed (HR from 1.35 to 2.16), while there was no change in the HR of history of chemotherapy (HR from 2.4 to 2.34).

Table 4 shows the multivariable analysis results for patients with limited BMs. While HRs of KPS and control of primary tumor increased markedly from the first to second period (from 1.14 to 3.16 and from 0.86 to 4.06, respectively), HRs of SRT and history of chemotherapy decreased (from 2.8 to 1.34 and from 1.82 to 1.3, respectively).

**Table 4:** Multivariable analyses of factors affecting survival in patients with limited BMs (1–3 lesions and < 4 cm in max diameter)

Variable	Category	Multivariable analysis														
		Limited BMs														
		Entire period					1997–2013					2014–2018				
		*n* = 127					*n* = 87					*n* = 40				
		*n*	%	HR	95% CI	*p*-value	*n*	(%)	HR	95% CI	*p*-value	*n*	(%)	HR	95% CI	*p*-value
Age at time of BMs (years)	< 65	100	(78.7)	ref		0.51	69	(79.3)	ref		0.68	31	(77.5)	ref		0.52
	≥ 65	27	(21.3)	0.83	0.47–1.42		18	(20.7)	0.86	0.43–1.68		9	(22.5)	0.68	2.00–2.12	
KPS score	≥ 70	77	(60.6)	ref		0.06	56	(64.4)	ref		0.66	21	(52.5)	ref		0.013
	< 70	50	(39.4)	1.56	0.98–2.46		31	(35.6)	1.14	0.63–2.00		19	(47.5)	3.66	1.33–10.6	
Presence of extracranial metastases	Brain only	13	(10.2)	ref		0.64	10	(11.5)	ref		0.79	3	(7.5)	ref		0.41
	Brain and other sites	114	(89.8)	0.83	0.41–1.85		77	(88.5)	1.12	0.49–2.89		37	(92.5)	0.45	0.08–3.78	
Control of primary tumor	Controlled	112	(88.2)	ref		0.35	80	(92.0)	ref		0.76	32	(80.0)	ref		0.04
	Uncontrolled	15	(11.8)	1.4	0.68–2.62		7	(8.0)	0.86	0.49–2.90		8	(20.0)	4.06	1.08–13.8	
WBRT	Performed	56	(44.1)	ref		0.65	49	(56.3)	ref		0.63	7	(17.5)	ref		0.38
	Not performed	71	(55.9)	1.24	0.50–3.10		38	(43.7)	1.32	0.42–3.89		33	(82.5)	0.36	0.04–4.37	
SRT	Performed	70	(55.1)	ref		0.08	38	(43.7)	ref		0.09	32	(80.0)	ref		0.81
	Not performed	75	(59.1)	2.3	0.9–5.9		49	(56.3)	2.8	0.87–9.00		8	(20.0)	1.34	0.12–18.0	
Targeted therapy	Performed	46	(36.2)	ref		0.52	22	(25.3)	ref		0.09	24	(60.0)	ref		0.18
	Not performed	79	(62.2)	1.16	0.73–1.88		65	(74.7)	1.69	0.92–3.27		14	(35.0)	0.42	0.10–1.44	
History of systemic chemotherapy before BMs	None	24	(18.9)	ref		0.002	14	(16.1)	ref		0.11	10	(25.0)	ref		0.73
	Performed	103	(81.1)	2.5	1.38–4.63		73	(83.9)	1.82	0.89–4.04		30	(75.0)	1.3	0.32–5.64	

*BMs* Brain metastases, *CI* confidence interval, *HR* hazard ratio, *KPS* Karnofsky performance status, *SRT* stereotactic radiotherapy, *WBRT* whole-brain radiotherapy

## Discussion

The present study showed an increase in the number of patients with BMs from CRC treated with SRT and a decrease in the number of those treated with WBRT when comparing two periods, i.e., before and after 2014. In patients with limited BMs, the proportion of those treated with WBRT decreased markedly in the second period. The increase in proportion of patients treated with SRT in the second period might be attributed to publication of the JLGK study [12], which led to the acceptance of using SRT for up to 10 lesions, as well as the installation of a dedicated stereotactic radiotherapy unit in our hospital.

Multivariate modeling should start with defendable assumptions that can be based on background knowledge (i.e., from previous studies in the same field of research) and prior knowledge [16, 17]. Statistical methods which use factors as covariates only if they are statistically significant in univariate analysis may be considered an “inappropriate use of bivariable analysis to screen risk factors for use in multivariable analysis”[18]. For this reason, the prognostic factors examined in the present study were selected based on previous studies. First, KPS was selected because it is an indicator that reflects a patient’s physical function and ability to perform daily activities, similar to the Eastern Cooperative Oncology Group Performance Status Scale (ECOG-PS) [19]. While ECOG-PS evaluates on a scale of 0 to 5, KPS has a scale ranging from 1 to 100, allowing for higher precision. Moreover, in a number of metastatic brain tumor-related clinical trials, KPS, rather than ECOG-PS, was used to assess physical function [5–8, 12, 20, 21]. In a similar manner, we selected “presence or absence of metastasis to other organs” as in the aforementioned RPA and DS-GPA studies [5–8]. Several publications have reported that the number of metastatic regions and ECOG-PS are prognostic factors in Stage IV colon cancer [22, 23]. However, in the present study, we included metachronous Stage I-III colon cancer in addition to Stage IV colon cancer. For this reason, we considered it appropriate to select “KPS” and “presence or absence of extracranial metastasis” as variables in this study.

When considering the history of chemotherapy up to the diagnosis of BM, it might be reasonable to use the “presence or absence” of treatment, rather than the “number of chemotherapy regimens,” for the following reasons. First, older cases for which the number of chemotherapy regimens is unknown are included. Second, the type of available drugs varies across different time periods. Thus, the “number of administered regimens” and the “number of remaining unused regimens” are considered unsuitable as covariates. Magni et al. found that patients who received surgery had longer survival times than those who received only radiation or chemotherapy [24], and Jung et al*.* reported that patients with a low RPA class and those with less previous chemotherapy had a good prognosis [25]. The findings of these two studies are consistent with those of the present study, demonstrating the influence of chemotherapy history on the prognosis of BM in CRC.

The prognosis of BMs from CRC improved in the second period compared to the first period. There were likely two major reasons for this improvement: an increase in the number of patients treated with SRT and advances in systemic chemotherapeutic agents. For the entire study period, significant prognostic factors for BM were KPS, control of primary tumor, SRT, and history of chemotherapy. In the analysis by period, some of these remained stable prognostic factors, while others did not (Table 3). Factors that did not change between the two periods were SRT and history of chemotherapy, and those that changed were KPS and control of primary tumor. Despite the smaller number of cases in the second period compared to the first period, KPS and control of primary tumor were significant prognostic factors with increased HR only in the second period.

As mentioned above, the number of patients treated with SRT increased in the second period, and SRT was an independent prognostic factor for the overall study population. SRT was not a significant prognostic factor in the two-period analysis, which might be explained by the small number of patients in each period and confounding by volume-related factors (limited or extensive BM). However, the finding that the HR of patients who were not treated with SRT was larger in the second period suggests a benefit of SRT. When the multivariate analysis was restricted to limited BM (Table 4), which may have a smaller prognostic impact than extended BM, there was a decreasing trend for the HR of SRT (from 2.8 to 1.34) in the second period. This suggests that the availability of other treatment options such as systemic chemotherapy may have weakened the prognostic impact, especially for limited BMs from CRC.

HRs for history of chemotherapy remained unchanged between the first and second periods. Previous studies have shown that patients who received less chemotherapy before developing BMs from CRC survive longer [20, 25, 26], suggesting that remaining available systemic chemotherapy after the diagnosis of BMs might be related to better prognosis. Use of targeted therapy was consistently associated with a better prognosis across both periods. Targeted agents used for systemic chemotherapy of metastatic CRC have been introduced in Japan, starting with bevacizumab in 2007, cetuximab in 2008, panitumumab in 2010, and ramucirumab in 2016, increasing the number of options and contributing to the longer survival of CRC patients [27–29].

While the HR of the presence of extracranial metastases (from 1.82 to 0.62), which is a known prognostic factor for BMs, decreased in the second period, the HRs of KPS and primary tumor control, which are prognostic factors for systemic chemotherapy, increased. KPS has prognostic relevance not only for the treatment of BMs, but also for systemic chemotherapy. In the analysis restricted to limited BMs, KPS was a significant prognostic factor only in the second period (Table 4). A good KPS might be associated with the effects of systemic treatment rather than BMs. Control of primary tumor is also a known prognostic factor for systemic chemotherapy, and was a significant prognostic factor only in the second period in the limited BM analysis. Since the primary lesion can be considered an extracranial lesion, the extracranial lesion may have had a lower prognostic impact (HR, from 1.85 to 0.93) in the second period. Moreover, in the limited BM analysis, the HR for the presence of extracranial lesions decreased from 1.12 in the first period to 0.45 in the second period. These results suggest that better control of extracranial lesions may have contributed to recent improvements in the prognosis of patients with BMs from CRC. Thus, systemic chemotherapy may play an important role in treatment strategies for CRC patients with BMs whose treatment has been optimized by SRT.

SRT may contribute to the improved prognosis of CRC with BMs in several ways, including improved local tumor control and reduced treatment side effects. For instance, Schoeggl et al*.* reported that SRT for BMs from CRC resulted in a high local tumor control rate of 94% with few complications [30], thereby delaying the worsening of symptoms and maintaining the quality of life of patients. In another study, Elaimy et al*.* reported that stereotactic radiosurgery (SRS) alone resulted in increased survival and local tumor control compared to WBRT in specific patient groups [31]. In a study by Takahashi et al*.*, the exposure dose to normal brain tissue increased with the number of target tumors in SRS for multiple BMs, but the exposure dose was acceptable when there were less than 7 targets [32]. These studies suggest that SRS can be advantageous for minimizing radiation exposure to normal brain tissue and reducing side effects, enabling patients to undergo other treatments (e.g., systemic chemotherapy) which can lead to improvements in overall survival.

Systemic chemotherapy, including molecular-targeted drugs, is the mainstay for prolonging overall survival, but in the case of BM, palliative effects are also important. Although we did not investigate differences in symptom relief between irradiation of BM by SRT or WBRT or a combination of both, many studies have reported less cognitive decline when SRS is used alone. For instance, Chang et al. reported that patients treated with SRS plus WBRT were at a greater risk of a significant decline in learning and memory function compared with those who received SRS alone [33]. Similarly, Brown et al*.* reported that a decline in cognitive function was more frequent with WBRT than with SRS, with no difference in overall survival between the treatment groups [21]. In that study, the SRS-alone group showed a lower rate of decline in memory and verbal fluency, long-term cognitive function, and quality of life compared to the SRS + WBRT group.

This study has potential limitations. First, there is potential selection bias stemming from the retrospective study of a single institute and the fact that some patients who visited our hospital for treatment of BMs from other institutions were treated with a different strategy than what we provide at our hospital before the BM diagnosis. Second, the sample size was relatively small. However, to our knowledge, the present patient population with BMs from CRC for which there was adequate background information, including KPS, is one of the largest reported to date.

In conclusion, the increase in overall survival of patients with BMs from CRC seen since 2014 may be attributed to advances in chemotherapy and molecular-targeted drugs, as well as the spread of SRT as an established standard therapy for BMs.
